# Thanks to all those who reviewed for *Journal of Negative Results in Biomedicine* in 2014

**DOI:** 10.1186/s12952-015-0025-9

**Published:** 2015-03-06

**Authors:** Bjorn R Olsen, Bent Brachvogel

**Affiliations:** Department of Cell Biology, Harvard Medical School, 240 Longwood Avenue, Boston, MA 02115 USA; Department of Pediatrics and Adolescent Medicine, Experimental Neonatology, University of Cologne, Joseph-Stelzmann-Straße 52, D50931 Cologne, Germany

## Abstract

A peer-reviewed journal would not survive without the generous time and insightful comments of the reviewers, whose efforts often go unrecognized. Although final decisions are always editorial, they are greatly facilitated by the deeper technical knowledge, scientific insights, understanding of social consequences, and passion that reviewers bring to our deliberations. For these reasons, the Editor-in-Chief and Managing Editor of the journal warmly thank the reviewers whose comments helped to shape *Journal of Negative Results in Biomedicine*, for their invaluable assistance with review of manuscripts for the journal in Volume 13 (2014).

**Courtney Aldrich**

USA

**Praveen Ballabh**

USA

**Markus Biburger**

Germany

**Trevor Birmingham**

Canada

**Frederick Colbourne**

Canada

**Peter Crouch**

Australia

**Jean-Pierre David**

Germany

**Solange L. De Castro**

Brazil

**Chetana S. Deshpande**

India

**Julia Etich**

Germany

**Yoshiaki Furukawa**

Japan

**Kolja Gelse**

Germany

**Felipe Goñi-de-Cerio**

Spain

**Bettina Groetsch**

Germany

**Ulrike Harre**

Germany

**Gordon Howarth**

Australia

**Dirk Hubmacher**

USA

**Pan Jihong**

China

**Dimitrios Kanakis**

Greece

**Masashi Kanayama**

USA

**Shunsuke Kanbara**

Japan

**Andre Pascal Kengne**

South Africa

**Michio Kurosu**

USA

**Alexander Benedikt Leichtle**

Switzerland

**Roberto Marci**

Italy

**David McGhee**

UK

**Dirk Mielenz**

Germany

**Tetsuryu Mitsuyama**

Japan

**Evelyn Marielle Monninkhof**

Netherlands

**Jennifer Moylan**

USA

**Takashi Nagasawa**

Japan

**Edin Nevzati**

Switzerland

**Nikoletta Papadopoulou**

Germany

**Rui Prediger**

Brazil

**Haibo Qiu**

China

**Jaqueline Rinaldi**

Brazil

**Juan Rodriguez**

Argentina

**Celena Scheede-Bergdahl**

Canada

**Je Kyung Seong**

South Korea

**Vishnumurthy Shushrutha Hedna**

USA

**Mitsutoshi Tsukimoto**

Japan

**Stephanie Wohlgemuth**

USA

**Fumihiko Yoshino**

Japan

**Ti-Fei Yuan**

China

